# Identification of the promising Persian walnut (*Juglans regia* L.) genotypes among seedling‐originated trees

**DOI:** 10.1002/fsn3.2193

**Published:** 2021-02-25

**Authors:** Narjes‐Sadat Mirmahdi, Ali Khadivi

**Affiliations:** ^1^ Department of Horticultural Sciences Faculty of Agriculture and Natural Resources Arak University Arak Iran

**Keywords:** breeding, genetic resource, germplasm, kernel percentage, walnut

## Abstract

Considerable genetic diversity among the native populations of Persian walnut (*Juglans regia* L.) provides a great opportunity to identify genotypes with valuable traits. In the present study, morphological and pomological diversity assessments of 362 walnut seedling origin genotypes were performed to identify superior genotypes. Significant differences were observed among the genotypes investigated in terms of the evaluated characters. Nut weighted from 5.53 to 19.24 g with an average of 10.67. The range of kernel weight was 1.78–9.28 g with an average of 4.83. Kernel percentage in 107 out of 362 genotypes studied was more than 50.00%. Multiple regression analysis (MRA) showed that kernel percentage was associated with kernel weight, nut weight, kernel filled, nut width, and shell thickness. Principal component analysis (PCA) showed that the characters related to nut and kernel size were correlated with the first component (PC1). Hierarchical cluster analysis (HCA) showed that the genotypes were clustered into two major clusters. Based on the most important commercial characters considered by breeders to select ideal walnuts, 15 superior genotypes were selected and are recommended for cultivation in the orchards and also can be used in breeding programs.

## Graphical abstract

Based on the most important commercial characters, which are considered by breeders to select ideal walnut genotypes, 15 superior genotypes were selected and are recommended for cultivation in the orchards and also and can be used in breeding programs.

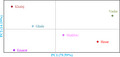

## 1 INTRODUCTION

The Persian walnut (*Juglans regia* L.) is monoecious, meaning that the male and female flowers are located separately on a tree (McGranahan & Leslie, 1990). This tree is self‐compatible, but most of the time, it shows protandry, and in some cases, protogyny (Mert, 2010), and because the period of pollen‐shedding does not entirely overlap female flower receptivity, so the cross‐pollination could be high in a particular condition.

Indigenous walnut populations have a significant genetic diversity because they are propagated through seed. Therefore, there is considerable variation in their phenological and carpological traits such as fruit size and shape, kernel thickness and color, kernel taste, and oil content (Korac et al., 1997; McGranahan & Leslie, 1990; Solar et al., 2002; Zeneli et al., 2004). In walnut breeding programs, essential goals such as selecting genotypes based on important commercial traits, including late‐leafing, late‐flowering, high kernel percentage, and lateral flowering habit are pursued (Zeneli et al., 2004).

Morphological descriptors are commonly used to examine the phenotypic diversity of germplasm (Bernard et al., 2018). So far, the first step in the description and grouping of germplasm, as well as the selection of superior genotypes in fruit trees, including walnuts, is morphological and pomological evaluations. One of the basic needs of germplasm management and its proper use in practical purposes of breeding is the accurate identification of genotypes (Solar et al., 2002). Morphological studies provide a guideline for selecting genotypes that are suitable for specific growth conditions. Morphological traits can be ideal for grouping walnut genotypes because they have considerable diversity and are also easy to use (Asadian & Pieber, 2005). Accordingly, the International Union for the Protection of New Varieties of Plants (UPOV, 1999) has provided guidelines for determining the differentiation, homogeneity, and stability of new cultivars in most plants (Solar & Stampar, 2011).

There is considerable diversity in the native populations of walnuts in Iran, due to propagation through seeds, high heterozygosity, and digocamy, and consequent cross‐pollination (Ebrahimi et al., 2015; Lansari et al., 2001). Significant genetic diversity provides an excellent opportunity to identify genotypes with valuable traits. Several studies have been done to identify and introduce the superior walnut genotypes (Jafari Sayadi et al., 2012; Hajnajari et al., 2012; Sarikhani et al., 2021) and also to investigate genetic diversity among populations of this species in Iran (Karimi et al., 2010; Mohsenipoor et al., 2010; Vahdati et al., 2015). In the present study, morphological and pomological diversity assessments of walnut seedling origin genotypes were performed to identify superior genotypes. Such information can help preserve important genetic resources and establish germplasm collections.

## 2 MATERIALS AND METHODS

### 2.1 Plant material

Morphological and pomological diversity assessments of 362 walnut seedling origin genotypes were performed to identify superior genotypes. The genotypes were selected from six areas of Shazand region in Markazi province/Iran, including Emarat, Ghale, Hesar, Khalaj, Shahbaz, and Vashe. Shazand region is located at 33˚87'35"N latitude and 49˚55'22"E longitude. The height of the selected areas ranged from 1958 to 2143 m above sea level. The studied genotypes were named based on the studied area. The selection of genotypes was based on commercial traits related to fruit. The age of the trees studied was not the same, but, several visits were done to select the trees with close ages.

### 2.2 The characters evaluated

Morphological assessments were done according to the walnut descriptor (IPGRI, 1994). The range of phenotypic diversity of genotypes was evaluated using 33 characteristics (Table 1). Fifteen characters were measured as continuous and the remaining were determined based on categorical scales. Samples of adult leaves were taken in summer from outside sections of trees. For fruit ripening date, when nuts of the first genotype were ripened, its date was recorded as zero. Then, the fruit ripening date of other trees was recorded based on it so that the number of days after the control or reference tree was considered as the ripening date of other trees. For the traits related to nut and kernel, 30 replicates were considered, and their mean was used for analysis. Nut and kernel dimensions were measured using a digital caliper (Anyi Instrument). Also, an electronic balance with the precision of 0.01 g (Ohaus SP602 AM, Fotronic Corporation) was used to measure the weight of nut and kernel. Kernel percentage was estimated using the “kernel weight/nut weight × 100” formula. The qualitative traits were evaluated based on the coding and scoring mentioned in the walnut descriptor (IPGRI, 1994), the list of which is shown in Table 2.

**TABLE 1:** Statistical descriptive parameters for morphological traits used to study walnut genotypes

No	Character	Abbreviation	Unit	Min	Max	Mean	SD	CV (%)^a^
1	Tree growth habit	TGH	Code	1	5	3.31	1.32	39.88
2	Tree growth vigor	TGV	Code	1	5	3.18	1.60	50.19
3	Tree height	TH	Code	1	5	3.64	1.46	40.11
4	Leaf color	LCo	Code	1	5	3.25	1.50	46.18
5	Leaf length	LLe	cm	24.12	53.58	34.98	4.39	12.54
6	Leaf width	LWi	cm	17.86	40.35	26.29	3.08	11.71
7	Petiole length	PLe	cm	13.75	34.67	20.19	3.39	16.78
8	Leaflet number	LtNo	Number	5.60	9.60	7.34	0.77	10.52
9	Terminal leaflet length	TeLtLe	cm	8.95	24.20	14.77	1.86	12.60
10	Terminal leaflet width	TeLtWi	cm	4.62	10.61	7.90	1.04	13.16
11	Terminal leaflet shape	TeLtSh	Code	1	5	3.17	1.42	44.83
12	Fruiting habit	FrHa	Code	1	5	1.10	0.48	44.00
13	Ripening date	RiDi	Date	06‐Sep	28‐Sep	3.43	1.39	40.55
14	Yield	Yi	Code	1	7	3.79	2.10	55.44
15	Nut length	NuLe	mm	27.08	44.71	34.91	3.12	8.95
16	Nut width	NuWi	mm	25.67	38.13	31.86	2.37	7.45
17	Nut weight	NuWe	g	5.53	19.24	10.67	2.19	20.49
18	Nut shape	NuSh	Code	1	13	5.61	3.20	57.11
19	Shell thickness	SheTh	mm	0.73	2.95	1.45	0.33	22.76
20	Shell hardness	SheHar	Code	1	7	3.05	2.14	70.13
21	Shell color	SheCo	Code	1	7	3.14	1.88	59.90
22	Shell seal	SheSe	Code	1	9	2.79	2.20	78.96
23	Shell surface serration	SheSu	Code	1	7	3.53	2.35	66.57
24	Kernel length	KeLe	mm	17.81	41.96	26.15	2.49	9.50
25	Kernel width	KeWi	mm	10.52	30.09	22.88	3.46	15.11
26	Kernel weight	KeWe	g	1.78	9.28	4.83	1.12	23.29
27	Kernel percentage	KePe	%	25.75	62.53	45.29	5.66	12.50
28	Kernel color	KeCo	Code	1	7	1.51	1.27	83.97
29	Kernel vein	KeVe	Code	1	7	3.02	1.73	57.15
30	Ease of kernel removal from nuts	EsKeRe	Code	1	7	1.90	1.55	81.74
31	Kernel filled	KeFi	Code	1	5	4.18	1.28	30.57
32	Kernel plumpness	KePl	Code	1	5	3.27	1.66	50.73
33	Kernel shriveling	KeShr	Code	1	5	3.40	1.64	48.12

Abbreviation: SD, Standard deviation.

^a^ CV=(*SD*/Mean) × 100.

**TABLE 2:** Frequency distribution for the measured qualitative morphological characters in the studied walnut genotypes

Character	Frequency (no. of genotypes)						
	1	3	5	7	9	11	13
Tree growth habit	Spreading (55)	Upright (196)	Extremely upright (111)	‐	‐	‐	‐
Tree growth vigor	Low (100)	Moderate (129)	High (133)	‐	‐	‐	‐
Tree height	Low (57)	Moderate (133)	High (172)	‐	‐	‐	‐
Leaf color	Light green (82)	Green (153)	dark green (127)	‐	‐	‐	‐
Terminal leaflet shape	Wide oval (77)	Oval (177)	Elliptic (108)	‐	‐	‐	‐
Fruiting habit	Terminal (346)	Mostly terminal (14)	Lateral (2)	‐	‐	‐	‐
Ripening date	Early (57)	Moderate (171)	Late (134)	‐	‐	‐	‐
Yield	Low (90)	Moderate (105)	High (101)	Very high (66)	‐	‐	‐
Nut shape	Round (68)	Board oval (49)	Oval (70)	Board ovate (93)	Ovate (58)	Trapezoidal (10)	Triangular (14)
Shell hardness	Paper (163)	Soft (66)	Moderate (94)	Hard (39)	‐	‐	‐
Shell color	Light (123)	Moderate (116)	Dark (98)	Very dark (25)	‐	‐	‐
Shell seal	Excellent seal (177)	Slightly open (99)	Moderate (43)	Wide (33)	Very wide (10)		
Shell surface serration	Low (139)	Moderate (63)	High (85)	Very high (75)	‐	‐	‐
Kernel color	Light (297)	Light amber (47)	Amber (8)	Brown (10)	‐	‐	‐
Kernel vein	Low (132)	Moderate (96)	High (133)	Very high (1)	‐	‐	‐
Ease of kernel removal from nuts	Easy (251)	Moderate (71)	Difficult (28)	Very difficult (12)	‐	‐	‐
Kernel filled	Low (30)	Moderate (88)	High (244)	‐	‐	‐	‐
Kernel plumpness	Low (103)	Moderate (107)	High (152)	‐	‐	‐	‐
Kernel shriveling	Low (92)	Moderate (106)	High (164)	‐	‐	‐	‐

### 2.3 Statistical analysis

Analysis of variance (ANOVA) was performed to evaluate the variation among the genotypes based on the traits measured using SAS software (SAS® Procedures, 1990). Simple correlations between traits were determined using Spearman correlation coefficients (SPSS Inc. Norusis, 1998). Principal component analysis (PCA) was used to investigate the relationship between genotypes and determine the main traits effective in genotype segregation using SPSS software. Hierarchical cluster analysis (HCA) was performed using Ward's method and Euclidean coefficient with PAST software (Hammer et al., 2001). The first and second principal components (PC1/PC2) were used to create a scatter plot with PAST software. Besides, independent traits affecting the kernel percentage as a dependent trait were determined through multiple regression analysis (MRA) using the “linear stepwise” method with SPSS software.

## 3 RESULTS AND DISCUSSION

### 3.1 Morphological and pomological description

The ANOVA (*p* < 0.01) revealed significant differences among the genotypes investigated in terms of the evaluated characters. The coefficient of variation (CV) ranged from 7.45 (in nut width) to 83.97% (in kernel color). The CV in 22 out of 33 characters was more than 20.00%, so that 11 characters, including kernel color, ease of kernel removal from nuts, shell seal, shell hardness, shell surface serration, shell color, kernel vein, nut shape, yield, kernel plumpness, and tree growth vigor, had the CVs more than 50.00% (Table 1).

Tree growth habit was predominantly upright (196 genotypes). Tree growth vigor and tree height were mainly high (133 and 172 genotypes, respectively) (Table 2). Leaf length ranged from 24.12 to 53.58 cm, leaf width varied from 17.86 to 40.35 cm, and petiole length ranged from 13.75 to 34.67 cm. Leaflet number ranged from 5.60 to 9.60, terminal leaflet length varied from 8.95 to 24.20 cm, and terminal leaflet width ranged from 4.62 to 10.61 cm (Table 1).

Terminal fruiting habit was predominant (346 genotypes), while fruiting was lateral only in two genotypes. Poggetti et al. (2017) reported that all walnut trees turned out to be terminal bearing in their survey. Previous studies in different countries have reported less than 5.00% of genotypes with lateral fruiting (Atefi, 1997; Botu et al., 2010; Korac et al., 1997; Solar et al., 2002). Only Rouskas & Zakynthinos (2001) reported that Greek walnuts were more laterally bearing in their study, but the trees they studied were a panel of selected genotypes.

Ripening date ranged from 06 to 28 September and was early in 57, moderate in 171, and late in 134 genotypes. The yield was highly variable, including low (90 genotypes), moderate (105), high (101), and very high (66). Also, nut shape showed strong variation and included round (68 genotypes), board oval (49), oval (70), board ovate (93), ovate (58), trapezoidal (10), and triangular (14) (Table 2).

Nut length ranged from 27.08 to 44.71 mm with an average of 34.91. The range of nut width was 25.67–38.13 mm with an average of 31.86 mm. Nut weighted from 5.53 to 19.24 g with an average of 10.67 (Table 1). The range of 6.00–16.89 g has been reported for nut weight in walnut from different countries (Ahandani et al., 2014; Cosmulescu, 2013; Ghasemi et al., 2012; Kabiri et al., 2018; Karadag & Akca, 2011). Akça et al. (2020) reported the range of 26.10–41.90 mm for nut length, 26.50 to 33.90 mm for nut diameter, and 6.21–15.18 g for nut weight in Kazakh walnuts. The minimum of 12.00 g for nut weight is accepted in walnut breeding programs (McGranahan & Leslie, 1990; Sharma & Sharma, 1998). Thus, 92 out of 362 genotypes studied had the nuts with more than 12.00 g.

Shell hardness was paper (163 genotypes), soft (66), moderate (94), and hard (39). Shell was excellently sealed in 177 genotypes and followed by slightly open (99 genotypes). More strength shells can better protect the kernels against the harmful organisms that feed on prevent the penetration of fungal pathogens (Korac et al., 1998). Shell thickness ranged from 0.73 to 2.95 mm and is similar to the ranges observed for this character in walnut from different countries (Akca et al., 2015; Kabiri et al., 2018; Karadag & Akca, 2011; Sharma et al., 2014). Generally, the most desirable shell thickness in walnut is 0.70–1.50 mm (Zhadan & Strukov, 1977). Thus, shell thickness in 185 out of 362 genotypes studied was lower than 1.50 mm. The ease of kernel removal from nuts is affected by shell thickness, and the quality of kernels is increased with the easier kernel removal from nuts from a commercial point of view (Sharma & Sharma, 1998).

Kernel color was predominantly light (297 genotypes). A primary object in walnut breeding programs is finding the genotypes with light kernel color (Kabiri et al., 2018). Kernel removal from nuts was easy in the majority of genotypes (251). The ease of kernel removal from nuts is an essential pomological feature. Korac et al. (1998) state that nuts with a thinner shell have a kernel easy to remove, especially if their shell is smooth, which is the most common case. Kernel plumpness was low (103 genotypes), moderate (107), and high (152). Kernel length ranged from 17.81 to 41.96 mm with an average of 26.15. Kernel width varied from 10.52 to 30.09 mm with an average of 22.88. The range of kernel weight was 1.78–9.28 g with an average of 4.83. Akça et al. (2020) reported the range of 2.36–6.64 g for kernel weight in Kazakh walnuts. The minimum of 6.00 g for kernel weight is accepted in walnut breeding programs (Germain, 1997; Korac et al., 1997; Sharma & Sharma, 1998). Thus, 53 out of 362 genotypes studied had kernels with more than 6.00 g.

**FIGURE 1:**
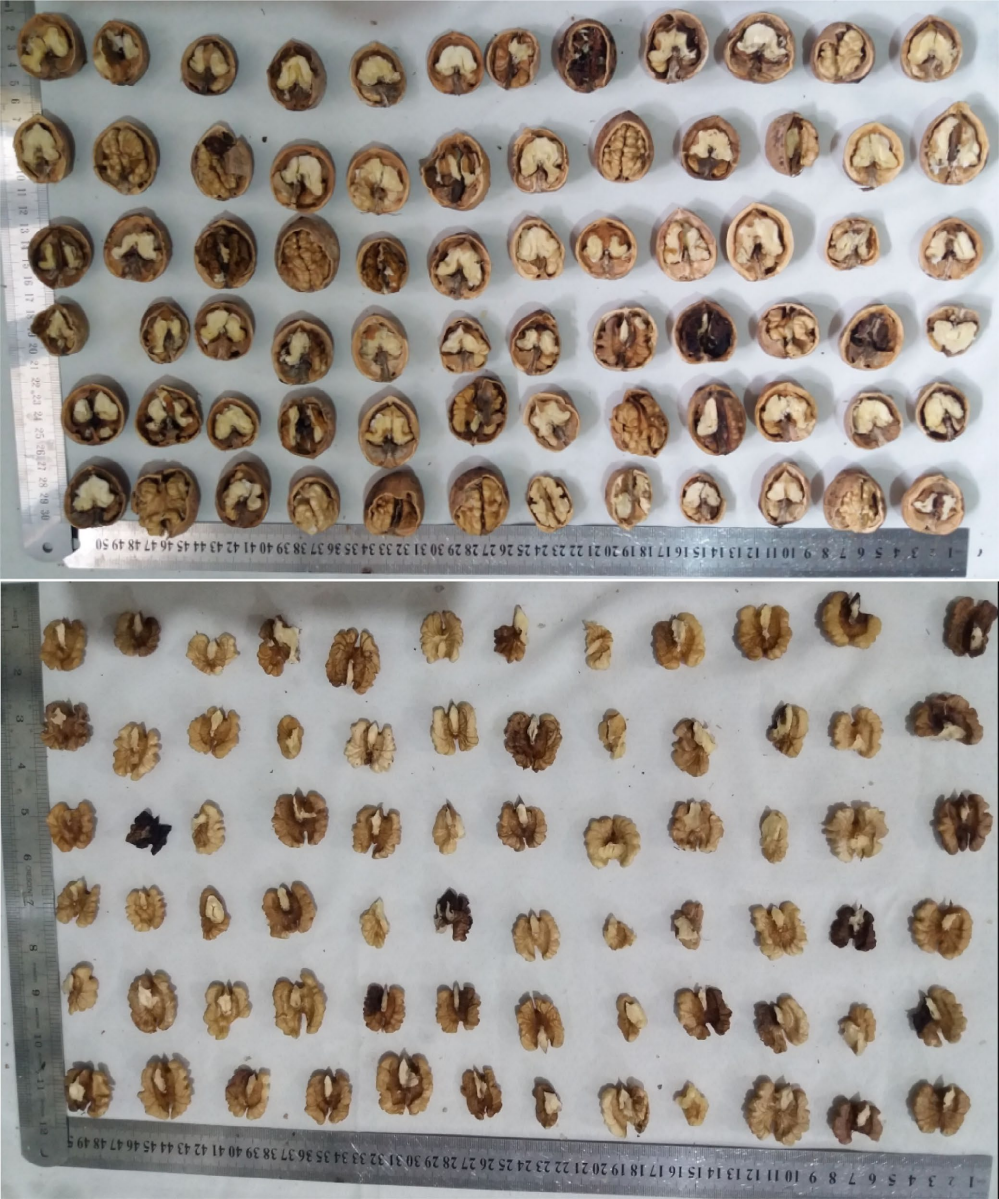
The pictures of some walnut genotypes studied pointing out diversity of nut and kernel‐related characters

Kernel percentage ranged from 25.75% to 62.53%, with an average of 45.29 (Table 1). The range of 33.55%–70.96% has been reported for kernel percentage in walnut from different countries (Ahandani et al., 2014; Akça et al., 2020; Kabiri et al., 2018; Karadag & Akca, 2011). The kernel percentage is a prominent character that is of great importance to walnut breeders (Cosmulescu & Botu, 2012). The kernel percentage is influenced by the weight of nut and kernel. The genotypes with a kernel percentage above 50.00% are preferred (Germain, 1997; Korac et al., 1998). Thus, kernel percentage in 107 out of 362 genotypes studied was more than 50.00%. The diversity of nut and kernel‐related characters is shown in Figure 1.

### 3.2 Correlations among the measured characters

Significant correlations were detected between important characters as revealed using Spearman correlation analysis (data not shown). Leaf length was positively and significantly correlated with leaf width (*r* = 0.72), petiole length (*r* = 0.90), leaflet number (*r* = 0.32), terminal leaflet length (*r* = 0.68), and terminal leaflet width (*r* = 0.48) and was in agreement with others (Sharma & Sharma, 1998; Khadivi‐Khub & Ebrahimi, 2015; Khadivi‐Khub et al., 2015a, b; Khadivi et al., 2019b). Yield showed positive and significant correlations with tree growth vigor (*r* = 0.37) and tree height (*r* = 0.17). Nut weight was positively and significantly correlated with nut length (*r* = 0.53) and nut width (*r* = 0.74). Shell hardness was positively and significantly correlated with shell thickness (*r* = 0.46). Kernel weight showed positive and significant correlations with nut length (*r* = 0.45), nut width (*r* = 0.68), nut weight (*r* = 0.83), kernel length (*r* = 0.59), and kernel width (*r* = 0.50), and agreed with others (Bayazit, 2012; Gahsemi et al., 2012; Cosmulescu & Botu, 2012; Mahmoodi et al., 2016; Poggetti et al., 2017; Khadivi et al., 2019b; Akça et al., 2020). Kernel percentage was negatively and significantly correlated with shell thickness (*r*= ‐ 0.30), shell hardness (*r*= ‐ 0.42), ease of kernel removal from nuts (*r*= ‐ 0.39), and kernel shriveling (*r*= ‐ 0.20), while it was positively and significantly correlated with kernel length (*r* = 0.17), kernel width (*r* = 0.30), kernel weight (*r* = 0.50), kernel filled (*r* = 0.46), and kernel plumpness (*r* = 0.41) and was in line with the previous findings (Sharma & Sharma, 1998; Cosmulescu & Botu, 2012; Khadivi et al., 2019a, 2019b; Khadivi‐Khub, 2014; Khadivi‐Khub et al., 2015a, 2015b.

### 3.3 Multiple regression analysis

The effect of independent traits on kernel percentage as a dependent trait was investigated with MRA (Table 3). The MRA showed that kernel percentage was associated with kernel weight, nut weight, kernel filled, nut width, and shell thickness. Thus, these key variables are the main traits accounting for kernel percentage, and they should be considered together in breeding with aiming increasing kernel percentage. Significant regression associations between kernel percentage and nut and kernel weights have been previously reported with MRA in walnut (Khadivi et al., 2019a; Khadivi‐Khub et al., 2015a, b).

**TABLE 3:** The traits associated with kernel percentage in the walnut genotypes as revealed by MRA and coefficients

Dependent character	Independent character	*r*	*r^2^*	Standardized beta coefficients	*t* value	*p* value
Kernel percentage	Kernel weight	0.499 a	0.25	1.73	70.84	0.00
	Nut weight	0.975 b	0.95	−1.46	−58.44	0.00
	Kernel filled	0.977 c	0.95	0.06	4.48	0.00
	Nut width	0.977 d	0.96	−0.05	−2.82	0.01
	Shell thickness	0.977 e	0.96	−0.03	−2.07	0.04

### 3.4 Principal component analysis

The PCA showed that the first 11 components accounted for 68.21% of the total variance (Table 4). The characters, including nut length, nut width, nut weight, kernel length, and kernel weight, were correlated with PC1, accounting for 11.38% of the total variance, called fruit size. The PC2 included leaf length, leaf width, terminal leaflet length, and terminal leaflet width, explaining 9.96% of the total variance called leaf size. Three characters, including shell thickness, shell hardness, and ease of kernel removal from nuts, formed the PC3, accounting for 7.57% of the total variance. In the previous studies, PCA has been used to investigate the phenotypic diversity of walnut genotypes (Cosmulescu & Stefanescu, 2018; Ebrahimi et al., 2015; Ghasemi et al., 2012; Hussain et al., 2016; Khadivi et al., 2019b; Khadivi‐Khub et al., 2015a, b; Norouzi et al., 2013; Rezaei et al., 2018).

**TABLE 4:** Eigenvalues of the principal component axes from the PCA of the morphological characters in the studied walnut genotypes

Character	Component										
	1	2	3	4	5	6	7	8	9	10	11
Tree growth habit	0.00	−0.08	0.02	−0.09	0.05	−0.14	0.75**	−0.08	−0.21	0.05	−0.03
Tree growth vigor	−0.01	−0.05	−0.02	0.07	−0.05	0.81**	−0.10	−0.08	0.07	0.08	−0.11
Tree height	0.05	0.15	−0.03	−0.01	0.04	0.16	0.78**	0.09	0.01	0.04	0.04
Leaf color	0.01	0.22	0.02	0.08	0.34	−0.09	−0.15	0.07	−0.13	0.44	−0.14
Leaf length	0.01	0.70**	−0.06	0.04	0.66	0.02	0.00	0.00	0.10	0.01	0.05
Leaf width	−0.01	0.76**	0.01	0.08	0.38	−0.05	0.02	−0.04	0.07	0.00	−0.08
Petiole length	−0.01	0.39	−0.10	0.03	0.82**	0.03	−0.01	0.01	0.08	−0.02	0.10
Leaflet number	−0.01	−0.21	−0.05	−0.10	0.82**	0.01	0.13	0.00	−0.10	−0.04	0.00
Terminal leaflet length	0.03	0.91**	0.03	0.04	0.06	−0.01	0.01	−0.01	0.10	0.06	−0.06
Terminal leaflet width	0.14	0.87**	−0.03	0.00	−0.17	0.06	0.03	0.05	−0.08	−0.03	0.10
Terminal leaflet shape	−0.10	−0.51	0.08	0.09	0.34	−0.05	−0.11	−0.02	0.26	0.06	−0.34
Fruiting habit	0.00	0.18	0.01	−0.04	0.01	0.10	−0.19	0.23	−0.29	−0.32	−0.33
Ripening date	−0.03	0.14	0.11	0.02	−0.06	−0.08	−0.06	−0.01	−0.11	0.32	0.59
Yield	0.10	0.07	0.08	0.00	0.05	0.79**	0.11	0.02	−0.05	−0.03	0.08
Nut length	0.79**	0.06	0.02	−0.08	0.00	0.08	0.04	0.02	0.04	0.29	−0.10
Nut width	0.81**	−0.02	−0.08	0.06	0.01	0.03	−0.08	0.02	0.02	−0.27	0.12
Nut weight	0.85**	0.10	0.26	0.23	−0.03	0.04	0.00	−0.06	0.03	−0.16	0.09
Nut shape	−0.06	−0.06	−0.06	−0.04	−0.10	0.11	0.13	0.10	0.05	0.74**	0.08
Shell thickness	0.06	−0.05	0.74**	0.05	−0.04	−0.05	0.09	0.04	−0.09	0.05	0.13
Shell hardness	−0.01	0.04	0.86**	−0.11	−0.01	0.03	−0.08	0.03	0.05	−0.03	0.00
Shell color	0.06	0.08	−0.04	0.03	−0.04	−0.04	−0.12	0.18	0.67**	−0.08	0.12
Shell seal	−0.01	−0.03	0.07	0.02	0.13	0.06	0.04	−0.01	0.28	−0.22	0.67**
Shell surface serration	0.29	−0.05	0.03	−0.20	0.06	0.17	−0.19	0.03	0.52	0.18	0.00
Kernel length	0.80**	0.05	−0.12	0.09	−0.02	0.00	0.12	0.01	0.06	0.14	−0.13
Kernel width	0.55	0.07	−0.50	0.15	0.08	−0.03	−0.06	−0.02	0.13	−0.08	0.09
Kernel weight	0.75**	0.06	−0.04	0.53	−0.06	0.00	0.01	−0.01	0.02	−0.11	0.00
Kernel percentage	0.02	−0.03	−0.46	0.65	−0.07	−0.06	0.01	0.06	0.00	0.05	−0.16
Kernel color	−0.04	−0.04	−0.04	−0.05	−0.12	−0.16	−0.04	0.68**	0.01	0.03	−0.08
Kernel vein	0.00	0.06	0.05	−0.01	0.13	0.09	0.13	0.72**	0.30	0.07	0.01
Ease of kernel removal from nuts	−0.09	−0.02	0.78**	−0.23	−0.07	0.07	−0.06	−0.06	0.06	−0.09	0.01
Kernel filled	0.20	0.05	−0.07	0.84**	0.02	0.05	−0.05	−0.04	−0.02	0.02	0.08
Kernel plumpness	0.19	0.04	−0.08	0.82**	0.01	0.06	−0.06	−0.12	−0.02	−0.03	0.04
Kernel shriveling	0.11	−0.02	0.07	−0.29	0.16	0.13	−0.23	0.48	−0.37	0.11	0.24
Total	3.75	3.29	2.50	2.42	2.29	1.48	1.47	1.37	1.34	1.31	1.29
% of Variance	11.38	9.96	7.57	7.35	6.95	4.49	4.45	4.16	4.05	3.96	3.89
Cumulative %	11.38	21.34	28.91	36.26	43.20	47.69	52.15	56.30	60.35	64.31	68.21

** Eigenvalues ≥ 0.67 are significant at the *p* ≤ 0.01 level.

The scatter plot created using PC1/PC2 showed phenotypic variations among the genotypes (not shown). Starting from negative to positive values of PC1, the genotypes showed gradual increases in nut length, nut width, nut weight, kernel length, and kernel weight. Also, starting from negative to positive values of PC2, the characters, including leaf length, leaf width, terminal leaflet length, and terminal leaflet width, showed gradual increases among the genotypes studied.

### 3.5 Hierarchical cluster analysis

**FIGURE 2:**
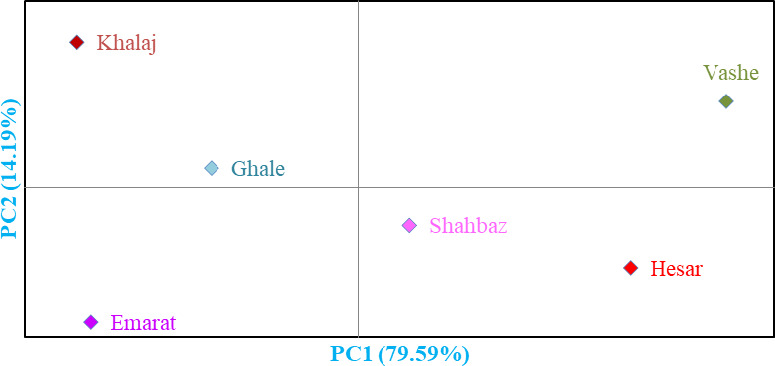
Biplot for the studied populations of walnut based on the morphological characters

The genotypes were clustered into two major clusters by the Ward dendrogram (not shown). The first cluster (I) was divided into two subclusters. Subcluster I‐A included 36 genotypes, while 81 genotypes were grouped into subcluster I‐B. The remaining genotypes were placed into the second cluster (II), forming three subclusters. Also, population analysis showed that the studied areas were divided into four main groups (Figure 2). Group I included Vashe area, while group II consisted of Khalaj and Ghale areas. Besides, group III included Shahbaz and Hesar areas, while group IV consisted of Emarat area.

The obtained results showed considerable phenotypic diversity in the Iranian walnut germplasm compared with that of other countries. This variability may be due, first, to genotypic variation or environmental conditions (Ghasemi et al., 2012). Second, walnuts species are monoecious and heterodichogamous, favoring outcrossing over selfing (Ebrahimi et al., 2015). Third, propagation by seeds of this species causes a significant genetic variability that appears at the flowering period for pomological characters, tree vigor, and type of fructification, which allow each geographic region to maintain a diverse population (Lansari et al., 2001). Morphological traits have been used effectively in detecting genetic variation on walnut in various studies (Cosmulescu & Stefanescu, 2018; Ebrahimi et al., 2015; Ghasemi et al., 2012; Hussain et al., 2016; Khadivi et al., 2019b; Khadivi‐Khub et al., 2015a, b; Norouzi et al., 2013; Rezaei et al., 2018).

In the breeding programs, for selection of an ideal walnut genotype, breeders should consider the most important commercial characters, including nut weight (≥12.00 g), kernel weight (≥6.00 g), kernel percentage (≥50.00%), shell thickness (0.70–1.50 mm), easier kernel removal from nuts, clean, strong, and thin shell, and light, clean, and plump kernel (Aslantas, 2006; Cosmulescu & Botu, 2012; Khadivi et al., 2019a, b; Khadivi‐Khub et al., 2015a, b; McGranahan & Leslie, 1990; Poggetti et al., 2017; Sharma & Sharma, 1998). Thus, 15 genotypes with having the threshold of the above characters were selected (Table 5).

**TABLE 5:** The most important fruit‐related traits of the superior walnut genotypes selected

Genotype	Nut length (mm)	Nut width (mm)	Nut weight (g)	Shell thickness (mm)	Shell hardness	Shell seal	Kernel length (mm)	Kernel width (mm)	Kernel weight (g)	Kernel percentage (%)	Kernel color	Ease of kernel removal from nuts	Kernel plumpness
Shahbaz‐1	37.00	35.77	16.39	0.75	Paper	Slightly open	29.84	27.44	8.98	54.81	Light	Easy	High
Shahbaz‐103	35.26	38.13	15.88	0.75	Paper	Excellent seal	28.13	29.27	8.18	51.49	Light	Easy	High
Shahbaz‐144	39.07	35.48	14.71	0.81	Paper	Slightly open	29.41	28.75	7.91	53.80	Light	Easy	High
Shahbaz‐170	34.48	35.55	12.82	0.87	Paper	Excellent seal	29.14	29.38	7.17	55.88	Light	Easy	High
Shahbaz−201	34.89	36.76	13.86	0.73	Soft	Excellent seal	28.38	28.86	7.00	50.53	Light	Easy	High
Vashe‐5	36.51	33.91	12.42	0.79	Paper	Excellent seal	27.82	19.38	6.74	54.24	Light	Easy	High
Shahbaz‐6	37.81	34.07	13.23	0.91	Paper	Slightly open	30.18	28.10	6.67	50.40	Light	Easy	High
Shahbaz‐60	41.95	33.89	12.49	1.01	Paper	Excellent seal	31.60	27.08	6.58	52.64	Light	Easy	High
Emarat‐6	35.27	33.42	12.91	1.21	Paper	Excellent seal	28.41	25.10	6.45	50.90	Light	Easy	High
Khalaj‐19	35.33	34.62	12.10	1.32	Paper	Excellent seal	28.06	26.73	6.43	53.15	Light	Easy	High
Khalaj‐11	35.98	34.42	12.61	1.33	Paper	Excellent seal	26.44	26.26	6.33	50.19	Light	Easy	High
Shahbaz‐29	37.77	32.37	12.35	0.79	Paper	Excellent seal	29.21	26.38	6.25	50.60	Light	Easy	High
Shahbaz‐75	36.55	33.41	12.03	0.83	Paper	Excellent seal	27.51	26.57	6.15	51.10	Light	Easy	High
Shahbaz‐209	39.30	31.21	12.13	0.86	Paper	Excellent seal	29.50	25.03	6.11	50.35	Light	Easy	High
Khalaj‐13	37.04	31.96	12.09	0.92	Paper	Excellent seal	26.60	25.71	6.05	50.02	Light	Easy	High

## 4 CONCLUSION

Walnut seeding‐originated trees form the structure of breeding programs both in terms of plant resources and production. Genetic diversity in plants, including walnuts, is increased and maintained through seeds. Therefore, seedling walnuts are valuable sources of the gene pool. The present investigation provided important information about walnut diversity based on different traits that can be used in selecting superior genotypes for various breeding or commercial purposes in the future. Based on the most important commercial characters considered by breeders to determine ideal walnut genotypes, such as nut weight, shell thickness, shell hardness, shell seal, kernel weight, kernel percentage, kernel color, and ease of kernel removal from nuts, 15 superior genotypes, including Shahbaz‐1, Shahbaz‐103, Shahbaz‐144, Shahbaz‐170, Shahbaz‐201, Vashe‐5, Shahbaz‐6, Shahbaz‐60, Emarat‐6, Khalaj‐19, Khalaj‐11, Shahbaz‐29, Shahbaz‐75, Shahbaz‐209, and Khalaj‐13, with having the threshold of the above characters, were selected that are recommended for cultivation in the orchards and also can be used in breeding programs.

## CONFLICT OF INTEREST

The authors declare no conflict of interest.

## RESEARCH INVOLVING HUMAN PARTICIPANTS AND/OR ANIMALS

None.

## INFORMED CONSENT

None.
